# Virtual reality clinical-experimental tests of compassion treatment techniques to reduce paranoia

**DOI:** 10.1038/s41598-020-64957-7

**Published:** 2020-05-22

**Authors:** Poppy Brown, Felicity Waite, Aitor Rovira, Alecia Nickless, Daniel Freeman

**Affiliations:** 1 Department of Psychiatry, University of Oxford, Oxford, UK; 2 Oxford Health NHS Foundation Trust, Oxford, UK; 3 School of Chemistry, University of Bristol, Bristol, UK

**Keywords:** Psychology, Human behaviour

## Abstract

Paranoia may build on negative beliefs held both about the self and others. Compassionate imagery may be one way of reducing such negative beliefs, and hence paranoia. Two studies tested this idea, one targeting compassion for the self and one targeting compassion for others. Two-hundred individuals from the general population scoring highly for paranoia were recruited. The studies used a randomised controlled experimental design, with embedded tests for mediation. Study one targeted self-compassion via creation of a compassionate coach (CC) image. Study two targeted compassion for others via loving kindness meditation (LKM). Individuals repeatedly entered neutral virtual reality social environments. Changes in compassion and paranoia were assessed. Compared to controls, the CC group increased in self-compassion (group difference = 2.12, C.I. = 1.57;2.67, *p* = <0.0001, *d* = 1.4) and decreased in paranoia (group difference = −1.73, C.I. = −2.48; −0.98, *p* = <0.0001, *d* = 0.8). Change in self-compassion explained 57% of change in paranoia. Compared to controls, the LKM group increased their compassion for others (group difference = 3.26, C.I. = 2.72;3.80, *p* = <0.0001, *d* = 1.7), and decreased in paranoia (group difference = −1.70, C.I. = −2.50; −0.89, *p* = <0.0001, *d* = 0.8). Change in compassion for others explained 67% of change in paranoia. Targeting negative beliefs about the self and others using compassionate imagery causes reductions in paranoia. Tests in clinical populations are indicated.

## Introduction

Treatments for paranoia (unfounded ideas of harm from others) need considerable improvement. Our approach to improvement is translational. We manipulate key mechanisms identified from our theoretical model and measure the effect on paranoia. When the manipulation reduces a key mechanism it can also inform treatment development. This is called an interventionist-causal model approach^1^. We substantiate the effects of the manipulation with mediation analysis for change^2^. The studies we report in this paper test the effects of compassion interventions in individuals from the general population scoring highly for current paranoid ideation. Paranoid thoughts have consistently been shown to exist on a spectrum of severity in the population; many people have a few paranoid thoughts and a few people have many^3–6^. The most severe form of paranoid ideation, known as persecutory delusions, lies at one end of this continuum and builds upon common emotional concerns^5^. The whole spectrum of paranoid experiences share an underlying aetiology, supported by evidence of a consistent heritability between mild and severe paranoia^7^. It is therefore possible to learn about clinical extremes by studying individuals with lower levels of severity.

Negative beliefs about the self and about others are highly correlated with clinical and non-clinical levels of paranoia^8^. It is hypothesised that such beliefs lead to feeling inferior, apart, and vulnerable, and that paranoia builds upon such concerns^9^. Compassionate interventions have started to be used to target negative beliefs^10,11^, and thus paranoia^12,13^. Self-compassion is strongly inversely correlated with negative ideas about the self^14^, and with severity of positive and negative symptoms in schizophrenia^15,16^. Training in self-compassion – e.g. through creating a compassionate coach (CC) image – can therefore help to reduce negative self-beliefs and moderate feelings of threat^13^. Similarly, training in compassion for others – e.g. through loving kindness meditation (LKM) – increases positive beliefs about others and so may enable individuals with paranoia to learn to see other people as a source of safety, rather than threat^17^.

In the first experimental test of compassion in relation to paranoia, Lincoln *et al*. (2012) found that practising compassionate coach imagery reduced paranoia in comparison to using neutral imagery (*d* = 0.59)^13^. We conducted two linked, randomised controlled tests of compassion interventions to reduce paranoia that built upon this experiment in three ways. First, we tested individuals reporting significant current paranoia. Second, we used immersive virtual reality (VR) to present participants with neutral social situations, and thus the opportunity to potentially form genuine paranoid ideation, which cannot be guaranteed by other means. Third, participants repeatedly entered different VR social experiences, enabling repeated measurement of key variables and thus temporal tests within the mediation analysis.

VR is increasingly being used in mental health research and treatment since individuals are typically more willing to enter the situations they find challenging in VR, and try out therapeutic techniques, because they know it is only a simulation^18^. VR has been used for treating patients with psychosis^19–21^, as well as for research with individuals from the general population reporting paranoia^22–24^. These studies have clearly demonstrated that entering neutral VR environments provokes paranoid ideation in vulnerable individuals. Thus VR provides a way of exposing, with a high level of control, individuals to situations that they find challenging.

Study one hypothesised that compared to a control group: those who generated a CC would experience increased self-compassion and decreased paranoid ideation in VR. Further, this decreased paranoia would be mediated by increased self-compassion. Similarly, study two hypothesised that compared to the control group: those who practised LKM would experience increased compassion for others and decreased paranoia. Further, decreased paranoia would be mediated by increased compassion for others. Our focus was at a clinical intervention level of causal explanation (an interventionist-causal approach): testing the effect of clinical techniques on the main hypothesised mechanisms (self-compassion in study one, compassion for others in study two) and the key psychological outcome (paranoia). We did not set-out to establish further detail in the causal chain (e.g. how alterations in compassion may affect other psychological processes).

## Study 1

### Method

Ethical approval was received from the Central University Research Ethics Committee (CUREC) at the University of Oxford and the study was performed in accordance with relevant guidelines and regulations. Informed consent was obtained from all participants.

### Participants

Participants were primarily recruited via social media and radio advertisements in Oxfordshire, UK. 740 participants were screened using questionnaires administered through Qualtrics. Exclusion criteria were: aged under 18 years; history of severe mental illness; photo-sensitive epilepsy; or self-identifying as having any significant visual, auditory, or mobility impairment. One-hundred individuals reporting six or more paranoid thoughts in the last month (a total score of 22 or above on the Green Paranoid Thoughts Scale Part B^25^ (GPTS-B) took part. This cut off score captures the upper quartile of paranoia scores in the general population^26,27^.

### Design

**Figure 1:**
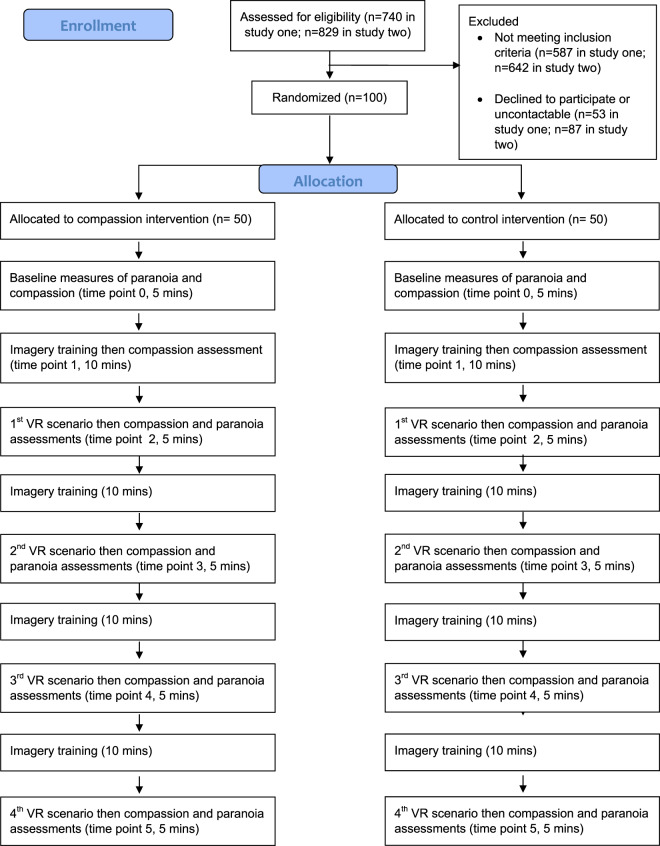
Experimental Procedure.

The design was between-groups. Each participant was tested in a single one-hour session. Participants completed baseline measures and were then randomised to the compassion or control condition. Randomisation was carried out using an online generator by an independent researcher. There were four stages of imagery development and four periods in VR social environments. Two different VR scenarios were used (a tube train and a lift), each experienced twice. Figure 1 shows the experimental procedure.

### Imagery interventions

We chose to train participants in creating an ideal compassionate coach unique to them. The coach provides strength, kindness, and warmth in order to help individuals feel better able to cope with everyday challenges. The aim is to harness the experience of being nurtured and eventually internalise it, so that a new and distinctive memory is created that can be easily accessed. A compassionate coach can have any identity, but must embody all the qualities of compassion, including strength, warmth, wisdom and kindness, and should encourage the individual to be kind toward them-self. A script was devised combining elements from a number of existing scripts^28–30^ that aimed to make the task more accessible and easy to achieve. The image was developed in four stages. First, participants created their coach and focussed on their qualities, before bringing to mind a difficult situation from their life, and practising having their coach help them to cope with it. For instance, the experimenter explained:

“Spend some time with your compassionate coach; they are there just for you, to comfort you and soothe you in any time of distress. They have your best interests at heart. They are someone who cares about you and strengthens your confidence; who makes you feel like you can face all of life’s challenges…With your coach there, you are not alone. You have someone with you, alongside you, and able to help you face this situation.”

Each latter stage allowed for greater detail and other (e.g. sensory) aspects of the coach to be developed. In between each of the four stages participants entered a VR social situation, during which they practised being self-compassionate with support from their coach. The control condition was identical except the image generated was entirely neutral: a weather forecaster, and participants were not instructed to think about their image during the VR scenarios.

The two imagery tasks were designed to be as similar as possible, apart from the affect associated with the images. The CC was intended to be warm and supportive, whereas the weather forecaster was neutral and provided only a weather commentary. Participants were encouraged to relax and take deep breaths in both conditions.

### Assessments

#### Paranoia

At baseline participants completed the Green Paranoid Thoughts Scale - Part B^25^. This is a 16-item scale assessing ideas of persecution such as ‘I was convinced there was a conspiracy against me’ and ‘I was sure someone wanted to hurt me’ on a 1–5 scale (1 = not at all, 5 = totally). Scores can range from 16–80; higher scores reflect greater paranoia. The scale is well validated for use in both clinical and non-clinical samples^31^ and has strong concurrent validity with paranoia severity as assessed by clinical interviews and by controlled virtual reality tests^32,33^. Using item response theory analysis with over 10,000 individuals, the GPTS-B has been shown to demonstrate high reliability (*a* > 0.95) across both mild and severe ends of the paranoia spectrum^34^. Test-retest reliability has also been shown to be good, with an intra-class correlation coefficient of 0.81^25^.

Two visual analogue scales were averaged to form a state measure of paranoia used for analysis. They were: ‘Please mark on the line below how vulnerable you felt during the virtual reality scenario’ and ‘Please mark on the line below how much you felt under threat during the virtual reality scenario’. The scale ranged from ‘Not at all’ to ‘Extremely’. These were completed after being in each VR environment. Cronbach’s alpha measure of internal consistency for the scale was 0.83. Visual analogue scales were chosen due to their sensitivity to change. Paranoia as measured on such scales has been correlated with both GPTS scores and interviewer assessment of paranoia^32^.

#### Self-compassion

Two visual analogue scales were also averaged to form a state measure of self-compassion for analysis. These were: ‘Please mark on the line below how kind you are feeling towards yourself right now’ and ‘Please mark on the line below how compassionate you are being towards yourself right now’. As with the paranoia measures these ranged from ‘Not at all’ to ‘Extremely’. Cronbach’s alpha measure of internal consistency for the scale was 0.85.

### Virtual Reality

#### The VR system

The VR setup included a tracking system allowing participants to move freely in an area of 3 × 3m. Participants wore a consumer VR head-mounted display (HMD), an HTC Vive PRO, with a resolution of 1440 × 1600 pixels per eye and a field of view of 110 degrees. It was powered by a computer with an Intel i7 CPU, a Nvidia GeForce GTX1080 graphics card, and Windows 10 operating system. The HMD includes an integrated audio system.

#### VR scenarios

Two scenarios of approximately three minutes were used: an underground tube train ride and a lift. These scenarios were based on those used in Freeman *et al*.^20^. The aim was to provide environments, although programmed to be neutral, that individuals with paranoia may find, to a degree, challenging, and thus helpful to practise the technique in. No specific instructions were given about observing the avatars or exploring the environments. The tube had a total of either 12–13 people on the carriage with three people in the central area near the participant in the first exposure and four in the second. The lift had either three or four avatars in it, for the first and second exposures respectively. The presence of an additional avatar in each second exposure aimed to increase the intensity of the social situation and prevent participants re-entering an identical scenario.

### Analysis

The target sample size was 100 individuals randomised equally between the experimental (CC image) and control (neutral image) group. We wanted to be able to detect moderate to large effect sizes. To detect an effect size of 0.6 using two-tailed t-tests and 80% power a sample size of 45 per group would be required. The use of mixed effects models would also allow greater statistical power and therefore detection of somewhat smaller effect sizes.

We used a linear mixed effects regression model for each continuous outcome in order to account for the repeated measures at the four different time points. This addressed hypotheses one and two i.e. whether a relationship exists between condition (compassion or control) and self-compassion, and between condition (compassion or control) and paranoia. We calculated standardised effect sizes with Cohen’s d, dividing the treatment effect by the shared standard deviation at baseline. To test the mediation hypothesis we tested two models. Firstly, we determined the extent of mediation of paranoia at the final time point, by self-compassion and self-kindness also at the final time point, as this is when the compassion intervention was complete and thus at its strongest. As a check on the direction of the relationship, we also conducted a reversed mediation test, putting paranoia at the final time point as the mediator and self-compassion at the final time point as the outcome. Due to the concern of conducting cross sectional mediation models, we also determined the extent of mediation of paranoia at the final time-point, by self-compassion and self-kindness at the mid time point, when half of the compassion intervention had been completed. This was able to assess mediation across time. The approach used was similar to that of Baron and Kenny (1986)^35^ but used a linear mixed effects model at each step. Two separate linear mixed effects models showed that the intervention was correlated with the outcome, and with the mediator.

A third model then used the outcome as the response and both the intervention and mediator as covariates. Extracting the parameters as per Baron and Kenny enabled us to obtain the total, direct and indirect effects and also the percentage mediation. Baseline measures of outcome and mediator were included as covariates in all models. We used R version 3.4.2 for the statistical analysis. We opted to use this approach, as in Freeman *et al*. (2017)^36^, as opposed to the method of using an instrumental variable approach with two-stage least squares, because the latter methodology has not been updated to include repeated measurements from the same participants. Since we had up to four repeated measurements per participant per outcome, we wanted to take advantage of all this information by using linear mixed effects models. Although randomisation ensures that the estimate of the intervention effect on the mediator and on the total intervention effect on the outcome are not affected by unaccounted confounders, the effect of the mediator on the outcome (path b) may still be affected by confounding^37^. We accounted for this by including baseline levels of the mediator and outcome in each of the linear mixed effects models. 

### Results

Participants were predominantly males in their late twenties, working full or part time. The mean age of participants was 29 (range 18–55 years). The mean GPTS-B scores of 35 and 33 in the compassion and control groups respectively indicated a much higher level of paranoia than in most analogue samples (e.g. 24.2 in Atherton *et al*., 2016; 25.6 Freeman, Evans *et al*., 2014)^22,23^ and are over the cut off used for inclusion in some clinical trials for persecutory delusions (e.g. 29 in Garety *et al*., 2017)^38^. Table 1 presents a summary of participant demographic and baseline characteristics. There were no missing data.

**Table 1:** Baseline and demographic characteristics by randomisation group.

	Study one – self-compassion		Study two – compassion for others	
	Compassion (n = 50)	Control (n = 50)	Compassion (n = 50)	Control (n = 50)
Age (years), mean (SD)	28.7 (8.6)	29.4 (9.6)	26.9 (9.8)	28.5 (10.1)
Men/women, *n/n*	33/17	30/20	27/23	32/18
**Ethnicity**, ***n***				
White British/Irish	34	35	31	34
Non White British/Irish	16	15	19	16
**Employment status**, ***n***				
Unemployed	3	2	4	2
Full/Part-time employed	30	36	28	22
Student	17	12	17	26
Retired	0	0	1	0
GPTS Part B score at baseline, mean (SD)	35.4 (11.2)	32.6 (10.0)	30.5 (11.0)	32.5 (12.4)
Self-compassion and self-kindness score at baseline, mean (SD)	6.5 (1.8)	7.0 (1.5)	—	—
Compassion for others score at baseline, mean (SD)	—	—	5.1 (2.0)	5.4 (1.7)

#### Hypothesis 1: Effect of condition on self-compassion

**Figure 2:**
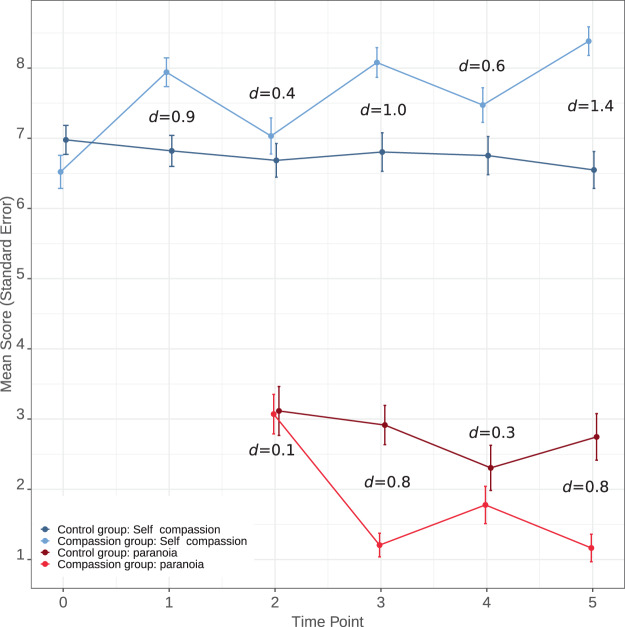
Scores and effect sizes for primary compassion and paranoia outcomes (study one).

Figure 2 shows the mean scores and effect sizes for the two outcomes at each time point. The compassion group showed significantly higher levels of self-compassion at all follow-up time points relative to the control group. For the final outcome the group difference was 2.12, 95% C.I. = 1.57;2.67, *p* = <0.0001, *d* = 1.4.

#### Hypothesis 2: Effect of condition on paranoia

The compassion group showed significantly lower levels of paranoia relative to the control group for both the mid and final time points. For the final outcome the group difference was −1.73, 95% C.I. = 2.48; −0.98, *p* = <0.0001, *d* = 0.8.

#### Hypothesis 3: Mediation

Table 2 shows the results from the mediation analysis. Change in self-compassion at the mid and final outcomes explained 36% and 57% respectively of the treatment effect on paranoia at the final outcome. In comparison, mediation analyses in the opposite direction indicated that changes in paranoia at the final time point explained only 24% of the change in self-compassion at the final time point.

**Table 2:** Total, direct, and indirect effect estimates from the mediation analysis.

	Study one		Study two	
	Self-compassion mid time point as mediator	Self-compassion final time point as mediator	Compassion for others mid time point as mediator	Compassion for others final time point as mediator
Total effect (CI, *P-*value)	−1.81 (−2.56; −1.07), <0.000	−1.81 (−2.56; −1.07), <0.000	−1.73 (−2.55; −0.92), <0.000	−1.73 (−2.55; −0.92), <0.000
Direct effect (CI), *P-*value	−1.17 (−1.95; −1.40), 0.003	−0.80 (−1.59; 0.00), 0.050	−1.14 (2.14; −0.14), 0.025	−0.58 (−1.67; 0.51), 0.297
Indirect effect (CI, *P-*value)	−0.65 (−1.08; 0.22), 0.001	−1.04 (−1.52; −0.56), <0.000	−0.58 (−1.18; −0.02), 0.057	−1.16 (−1.94; −0.38), 0.003
Percentage mediation	35.84	57.43	33.57	66.89

## Study 2

### Method

The recruitment method for a new cohort of 100 individuals and the study design were identical to study one. The content of the imagery intervention, however, targeted compassion for others as opposed to self-compassion. Accordingly, additional measures were included that assessed compassion for others.

Study two had several differences to study one. Firstly, the outcome measures used novel wording that had not been discussed in the compassion intervention. Secondly, a measure of positive affect was included meaning it could be ascertained whether this may also be a mediator of change in paranoia. Finally, at the end of the study participants in the compassion group were asked to describe how they found the compassion training so that we gained qualitative feedback.

### Imagery intervention

LKM uses visualisation, (e.g. imagining someone smiling at you) reflection (e.g. thinking about yours and others’ positive qualities), and auditory techniques (e.g. internally repeating phrases such as ‘I hope you have a good day’). A script was devised combining elements from a number of existing scripts and protocols^17,39^. As with study one there were four stages of training. The first stage asked individuals to imagine receiving and sending, warmth and love to one or more persons to whom they are very close. For instance, the experimenter explained:

“Reflect on their positive qualities…you could picture them being happy, maybe laughing with you”

“See if you can let yourself fill with warmth…maybe the flow of warmth is associated with a colour”

“You could repeat that you wish this person to feel happy, to have a nice day”.

Individuals were then asked to imagine themselves on a bus or train and to try and send warmth and kindness to some of the strangers around them on the bus or train, including the driver. Each of the other stages required practising with different people, including someone whom they disliked, a neutral acquaintance, and groups of family, friends, and strangers. The control condition was identical to study one. As in the previous study, participants entered a social situation in virtual reality in between each stage of imagery training, during which those in the compassion group were asked to further practise the exercise with the VR avatars.

### Assessments

#### Paranoia

Paranoia measures were identical to study one.

#### Compassion for others

Three visual analogue scales were averaged to form a measure of compassion for others used for analysis. At baseline and following the first imagery training session individuals were asked to imagine they were walking down a street before answering the analogue scales: ‘Please mark on the line below how connected you would feel to the people around you’, Please mark on the line below how understanding you would feel of the people around you’ and Please mark on the line below how accepting you would feel of the people around you’. Following each VR scenario the questions were based on how connected, how understanding and how accepting of the VR avatars participants felt. These measures again ranged from ‘Not at all’ to ‘Extremely’. Cronbach’s alpha measure of internal consistency for the scale was 0.9.

#### Positive Affect

A visual analogue scale similarly ranging from ‘Not at all’ to ‘Extremely’ asked participants: ‘Please mark on the line below how positive you feel right now’.

### Analysis

Analysis was identical to study one. An additional mediation analysis was also run that assessed positive affect at the final time point as a mediator.

### Results

Demographic and baseline characteristic were very similar to study one (see Table 1). One participant had missing data for the final three time points due to running out of time after finding the first two stages of compassion exercise particularly emotional.

#### Hypothesis 1: Effect of condition on compassion for others

**Figure 3:**
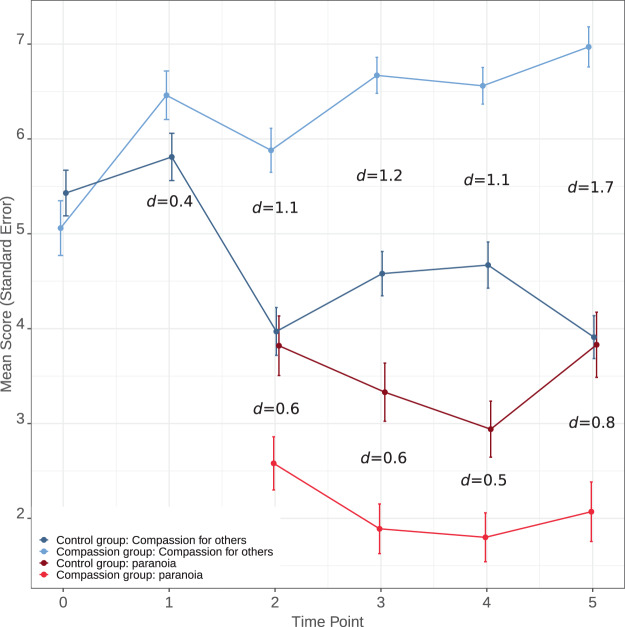
Scores and effect sizes for primary compassion and paranoia outcomes (study two).

Figure 3 shows the mean scores and effect sizes for the two outcomes at each time point. The compassion group showed significantly higher levels of compassion for others at all follow-up time points relative to the control group. For the final outcome the group difference was 3.26, 95% C.I. = 2.72;3.80, *p* = <0.0001, *d* = 1.7.

#### Hypothesis 2: Effect of condition on paranoia

The compassion group showed significantly lower levels of paranoia relative to the control group at all follow-up time points. For the final outcome the group difference was –1.70, 95% C.I. = −2.50; −0.89, p = <0.0001, *d* = 0.8.

#### Hypothesis 3: Mediation

Table 2 shows the results from the mediation analysis. Change in compassion for others at the mid and final outcomes explained 34% and 67% respectively of the treatment effect on paranoia at the final outcome. Mediation analysis in the opposite direction indicated that changes in paranoia at the final outcome explained just 4% of the change in self-compassion at the final time point. Change in positive affect at the final time point accounted for 47% of the change in paranoia at the final time point. Mediation in the opposite direction showed that change in paranoia accounted for 24% of the change in positive affect at final outcome.

## General Discussion

This paper draws on the hypothesis that paranoia builds upon negative ideas about the self and others. To our knowledge, these were the first randomised controlled experimental tests of compassion intervention techniques in individuals with current paranoid ideation. The interventionist-causal approach allows for inferences to be made concerning both treatment development and causal mechanisms^1^.

The first necessary stage in such an approach is for the treatment technique to alter the targeted mechanism, which was achieved. Both studies found large effect size increases in compassion at all time points. This demonstrates that even brief compassion interventions, when practised in VR social environments, are effective in increasing state compassion. There is therefore potential for both CC imagery and LKM to be tested as clinical techniques, perhaps with the aid of VR, in therapy for individuals with persecutory delusions. Anecdotally, participants reported finding it surprisingly easy to create their CC and found it useful to practise using their coach in VR social environments. Many based their coach on someone they knew, or on animals or trees, for example. Some chose inanimate shapes of a particular colour, and one individual used a perfected version of themselves. In study two participants also reported how useful they found the training:

“I felt kinder to myself by the end and tried to be kinder towards others”

“I think I’ll use this in situations where I don’t feel as comfortable”

“It felt more like I was in a safer place…the people weren’t just strangers”.

The success of the manipulations meant that the effects on paranoia could be determined. In both studies those who received the compassion intervention showed a large reduction in paranoia. Because of the manipulationist design of the studies (i.e. it was level of compassion that was targeted and altered) the inference is that low compassion for the self and for others is likely to be a contributory causal factor in the occurrence of paranoid thoughts. The mediation and reverse mediation analyses support this interpretation. Although study two found that increased positive affect may also be part of the causal pathway, the results suggest compassion was the stronger mediator. Moreover, the reversed mediation analysis of paranoia on positive affect was also quite large (24%) suggesting positive affect may act as a moderator rather than mediator.

It is interesting that effects were most evident at the middle and final time points. Particularly in study one there was a noticeable drop in compassion and increase in paranoia at the fourth time point. This is likely due to the fourth time point being the first experience of the VR lift scenario. The novelty of the scenario may have made it difficult for participants to employ their compassionate coach straight away. It is not clear why a similar drop was not seen in study two. In contrast, the middle and final time points were the second time participants experienced each VR scenario, with an additional avatar present in the scene. This perhaps increased the level of paranoia of the control groups, but not that of the compassionate groups, who could employ the compassionate imagery, which would have been established to a greater degree after the previous practicing.

There are a number of limitations to the studies. First, non-clinical samples were used meaning the results may not be generalisable to those experiencing more severe paranoia. Potential fears of compassion may first need to be investigated within clinical samples^40^. Moreover, each stage of imagery training would likely need to be extended to become its own session, with a gap of perhaps a week in between stages in order to allow consolidation of each training stage and further practise in daily life. Second, neither the researcher nor participants were blind to the intervention that participants were receiving, although participants were blind to the study hypotheses and the outcomes were self-report. Third, CC imagery and LKM are two of many compassion techniques. There may be other techniques, not tested here, that are better. Fourth, in study one the outcome measures used words that were included in the manipulation itself, i.e. self-compassion and kindness, which might mean increased identification with those words in the manipulation check was unsurprising. On the other hand, we tested a technique in the way that it would be administered in clinical practise: teaching patients about a concept, practising a technique to modify it, then asking if they feel improvement in it. Moreover, this issue was not present in study two, which used novel wording of outcome measures, yet similar effects were found. Fifth, there was no long term follow up. A follow up time point would have allowed us to assess mediation across time using the endpoint of the intervention as the mediator and follow up time point of paranoia as the outcome. The lack of follow up also means only the short-term effects of the manipulation were examined; it is unknown whether there were any lasting benefits. Given the briefness of the interventions we would not necessarily expect to see long-term effects. However, the results do show support for testing the interventions in patients with severe paranoia with investigation into longer-lasting effects (with consideration on how to embed the techniques into day-to-day life).

Finally, we explored only one level of causal explanation: that the techniques increased compassion and this impacted levels of paranoia. We did not set out to test the potential cascade of effects within this causal chain (e.g. by assessing other mechanisms or altering the control condition). Although study two assessed the impact of positive affect and found it to be a smaller mediator of change than compassion, it is always possible to further disentangle causal pathways, e.g. we could have also measured variables such as anxiety or attention. Similarly, compassionate coach imagery could be seen primarily as an exercise in receiving compassion rather than in self-compassion, but we did not measure this. We also cannot ascertain from these studies whether compassionate interventions work primarily at an affective level of whether they, for example, allow switching from social threat to affiliation processing systems. The findings from this study therefore open up a number of avenues for future research to discover greater knowledge about the causal chain.

## References

[1] Kendler KS, Campbell J (2009). Interventionist causal models in psychiatry: repositioning the mind–body problem. Psychol. Med..

[2] Dunn G (2015). Evaluation and validation of social and psychological markers in randomised trials of complex interventions in mental health: a methodological research programme. Health Technol. Assess..

[3] Bebbington PE (2013). The structure of paranoia in the general population. Br. J. Psychiatry.

[4] Wong KK, Freeman D, Hughes C (2014). Suspicious young minds: Paranoia and mistrust in 8- To 14-year-olds in the UK and Hong Kong. Br. J. Psychiatry.

[5] Freeman D (2005). Psychological investigation of the structure of paranoia in a non- clinical population. Br. J. Psychiatry.

[6] Bird JC, Waite F, Rowsell E, Fergusson EC, Freeman D (2017). Cognitive, affective, and social factors maintaining paranoia in adolescents with mental health problems: A longitudinal study. Psychiatry Res..

[7] Zavos HMS (2014). Consistent etiology of severe, frequent psychotic experiences and milder, less frequent manifestations: A twin study of specific psychotic experiences in adolescence Objective—Investigate degree of genetic and environmental influences on specific psychoti. JAMA Psychiatry.

[8] Fowler D (2006). The Brief Core Schema Scales (BCSS): Psychometric properties and associations with paranoia and grandiosity in non-clinical and psychosis samples. Psychol. Med..

[9] Freeman D (2016). Persecutory delusions: a cognitive perspective on understanding and treatment. The Lancet Psychiatry.

[10] Kirby JN, Tellegen CL, Steindl SR (2017). A Meta-Analysis of Compassion-Based Interventions: Current State of Knowledge and Future Directions. Behav. Ther..

[11] Hickey T, Nelson B, Meadows G (2017). Application of a mindfulness and compassion-based approach to the at-risk mental state. Clin. Psychol..

[12] Ascone L, Sundag J, Schlier B, Lincoln TM (2017). Feasibility and Effects of a Brief Compassion-Focused Imagery Intervention in Psychotic Patients with Paranoid Ideation: A Randomized Experimental Pilot Study. Clin. Psychol. Psychother..

[13] Lincoln TM, Felicitas H, Hartmann M (2012). Can Paranoid Thoughts be Reduced by Targeting Negative Emotions and Self-Esteem? An Experimental Investigation of a Brief Compassion-Focused Intervention. Cognit. Ther. Res..

[14] Collett N, Pugh K, Waite F, Freeman D (2016). Negative cognitions about the self in patients with persecutory delusions: An empirical study of self-compassion, self-stigma, schematic beliefs, self-esteem, fear of madness, and suicidal ideation. Psychiatry Res..

[15] Eicher AC, Davis LW, Lysaker PH (2013). Self-Compassion: A Novel Link With Symptoms in Schizophrenia?. J. Nerv. Ment. Dis..

[16] Mills A (2007). Paranoid Beliefs and Self-Criticism in Students. Clin. Psychol. Psychother. Clin. Psychol. Psychother.

[17] Hutcherson CA, Seppala EM, Gross JJ (2008). Loving-Kindness Meditation Increases Social Connectedness. Emotion.

[18] Freeman, D. *et al*. Virtual reality in the assessment, understanding, and treatment of mental health disorders. *Psychol. Med*. **47**, 2393-2400 10.1017/S003329171700040X (2017). 10.1017/S003329171700040X PMC5964457 28325167

[19] Pot-Kolder, R. *et al*. Effects of virtual reality based cognitive behavioural therapy for paranoid ideation and social avoidance in patients with a psychotic disorder: a randomised controlled trial. *The Lancet Psychiatry* **5**, 217–226, 10.1016/S2215-0366(18)30053-1 (2018). 10.1016/S2215-0366(18)30053-1 29429948

[20] Freeman D (2016). Virtual reality in the treatment of persecutory delusions: Randomised controlled experimental study testing how to reduce delusional conviction. Br. J. Psychiatry.

[21] Freeman D (2019). Automated virtual reality (VR) cognitive therapy for patients with psychosis: Study protocol for a single-blind parallel group randomised controlled trial (gameChange). BMJ Open.

[22] Atherton S (2016). Self-Confidence and Paranoia: An Experimental Study Using an Immersive Virtual Reality Social Situation. Behav. Cogn. Psychother..

[23] Freeman D (2014). Height, social comparison, and paranoia: An immersive virtual reality experimental study. Psychiatry Res..

[24] Freeman D (2003). Can Virtual Reality be Used to Investigate Persecutory Ideation?. J. Nerv. Ment. Dis..

[25] Green, C. E. L. *et al*. Measuring ideas of persecution and social reference: the Green et al. Paranoid Thought Scales (GPTS). *Psychol. Med*. **38**, 101–111 10.1017/S0033291707001638 (2008). 10.1017/S0033291707001638 17903336

[26] Freeman D, Lister R, Evans N (2014). The use of intuitive and analytic reasoning styles by patients with persecutory delusions. J. Behav. Ther. Exp. Psychiatry.

[27] Freeman D, Evans N, Lister R (2012). Gut feelings, deliberative thought, and paranoid ideation: A study of experiential and rational reasoning. Psychiatry Res..

[28] Gilbert P, Procter S (2006). Compassionate mind training for people with high shame and self-criticism: Overview and pilot study of a group therapy approach. Clin. Psychol. Psychother..

[29] Kolts, R. *The compassionate-mind guide to managing your anger: Using compassion-focused therapy to calm your rage and heal your relationships*. (New harbinger Publications, 2012).

[30] Welford, M. The Compassionate Mind Approach to Building Self-Confidence. (Constable & Robinson, 2012).

[31] Statham V, Emerson LM, Rowse G (2019). A systematic review of self-report measures of paranoia. Psychol. Assess..

[32] Freeman D (2014). The use of immersive virtual reality (VR) to predict the occurrence 6 months later of paranoid thinking and posttraumatic stress symptoms assessed by self-report and interviewer methods: a study of individuals who have been physically assaulted. Psychol. Assess..

[33] Freeman D, Pugh K, Vorontsova N, Antley A, Slater M (2010). Testing the continuum of delusional beliefs: An experimental study using virtual reality. J. Abnorm. Psychol..

[34] Freeman, D. *et al*. The revised Green *et al*. Paranoid Thoughts Scale (R-GPTS): psychometric properties, severity ranges, and clinical cut-offs. *Psychol. Med*. 1–10 10.1017/s0033291719003155 (2019). 10.1017/S0033291719003155 PMC7893506 31744588

[35] Baron RM, Kenny DA (1986). The Moderator-Mediator Variable Distinction in Social The Moderator-Mediator Variable Distinction in Social Psychological Research: Conceptual, Strategic, and Statistical Considerations. J. Pers. Soc. Psychol..

[36] Freeman D (2015). Effects of cognitive behavioural therapy for insomnia on the mental health of university students: study protocol for a randomized controlled trial. Trials.

[37] Whittle R, Mansell G, Jellema P, van der Windt D (2017). Applying causal mediation methods to clinical trial data: What can we learn about why our interventions (don’t) work?. Eur. J. Pain (United Kingdom).

[38] Garety PA (2017). SlowMo, a digital therapy targeting reasoning in paranoia, versus treatment as usual in the treatment of people who fear harm from others: study protocol for a randomised controlled trial. Trials.

[39] Salzberg, S. *Lovingkindness: The revolutionary art of happiness*. (Shambala Publications, 1995).

[40] Martins, M. J. *et al*. Recovery through affiliation: a Compassionate Approach to Schizophrenia and Schizoaffective Disorder (COMPASS). *J. Context. Behav. Sci*. **9**, 97–102 10.1016/j.jcbs.2018.07.009 (2018).

